# Elevated activated partial thromboplastin time-based clot waveform analysis markers have strong positive association with acute venous thromboembolism

**DOI:** 10.11613/BM.2019.020710

**Published:** 2019-06-15

**Authors:** Chuen Wen Tan, McVin Hua Heng Cheen, Wan Hui Wong, Ian Qianhuang Wu, Brian Lee Wei Chua, Sahul Hameed Ahamedulla, Lai Heng Lee, Heng Joo Ng

**Affiliations:** 1 Department of Hematology, Singapore General Hospital, Singapore; 2 Department of Pharmacy, Singapore General Hospital, Singapore

**Keywords:** blood coagulation, activated partial thromboplastin time, thrombophilia, thrombosis, venous thromboembolism

## Abstract

**Introduction:**

A hypercoagulable state is a predisposition for venous thromboembolism (VTE). The activated partial thromboplastin time (aPTT)-based clot waveform analysis (CWA) is a global haemostatic measure but its role in assessment of hypercoagulability and thrombotic disorders is uncertain. We aimed to study the changes of CWA parameters in acute VTE. We hypothesized that patients with acute VTE would demonstrate higher CWA values than control patients without VTE and having elevated CWA parameters is associated with acute VTE.

**Materials and methods:**

Clot waveform analysis data from patients (N = 45) with objectively proven acute VTE who had an aPTT performed prior to initiation of anticoagulation were compared with controls (N = 111). The CWA parameters measured were min1, min2, max2 and delta change.

**Results:**

While the mean aPTT between VTE patients and controls did not differ (P = 0.830), the mean CWA parameters were significantly higher among VTE patients than controls (min1, P < 0.001; min2, P = 0.001; max2, P = 0.002; delta change, P < 0.001). There were significantly more cases within the VTE group exhibiting CWA values above their reference intervals than the control group (all P < 0.001), with the odds ratios for VTE of 8.0, 5.2, 4.8 and 18.6 for min1, min2, max2 and delta change, respectively (all P < 0.001).

**Conclusions:**

Patients with acute VTE had elevated aPTT-based CWA parameters than controls. Higher CWA parameters were significantly associated with acute VTE.

## Introduction

Venous thromboembolism (VTE) is a common medical condition with an incidence rate of approximately 1 *per* 1000 person-years. It confers significant morbidity and mortality to those afflicted. The pathogenesis of VTE is multifactorial and the causative elements could be broadly categorized into 3 groups – alteration of blood flow, endothelial injury and hypercoagulability of the blood, as described in Virchow’s triad. Consistently, changes in coagulation system that lead to a hypercoagulable state are risk factors for formation of thrombosis. Elevated concentrations of clotting factors II, VIII, IX, XII, and fibrinogen have been demonstrated to be independent risk factors for VTE (*1*-*3*). Not surprisingly, a shortened activated partial thromboplastin time (aPTT) also confers a higher risk for deep vein thrombosis (DVT) (*4*-*7*). Similarly, global haemostatic measures of thrombin generation positively predict the risk for VTE (*8*-*12*).

Clot waveform analysis (CWA) is an alternate form of global haemostatic assessment that evaluates the clot formation kinetics within the routine clotting tests. To date, the majority of published works on its utility have focused on congenital bleeding disorders, disseminated intravascular coagulopathy and sepsis (*13*-*16*). Existing literature of CWA in thrombotic disorders is evidently lacking and it was not until recently that CWA has been investigated in this domain. Higher CWA parameters were shown to be associated with high Padua Prediction Score, a VTE risk assessment tool for hospitalized medical patients, suggesting utility as a surrogate for hypercoagulability (*17*). Activated partial thromboplastin time - based CWA may provide an alternative avenue to assess the risk or serve as a marker of thrombosis in thrombotic disorders, but more supporting evidence is keenly awaited. Thus, we aimed to study the changes of CWA parameters in patients with acute VTE. We hypothesized that acute VTE patients have increased aPTT-based CWA parameters and this elevation is associated with hypercoagulability and thus VTE. Herein, we report our findings on aPTT-based CWA parameters of patients with acute VTE compared with those without VTE. In addition, the diagnostic role of CWA in acute VTE was also evaluated.

## Materials and methods

### Study design

This retrospective cross-sectional study was carried out at Singapore General Hospital, Singapore, an academic tertiary medical centre with comprehensive haematology laboratory services. This study was approved by our Centralized Institutional Review Board with waiver of consent granted for retrospective retrieval of CWA and relevant clinical data (Protocol Reference: 2017/2169).

There were two main study groups consisting of patients with acute VTE and controls without VTE. Patients with acute VTE were actively managed by the Department of Hematology between April 2017 and October 2017, whereas the data-browsing period occurred between April 2017 and November 2017. The control group consisted of patients admitted for elective orthopedic and urological procedures between October 2015 and January 2016, with data browsing between November 2015 and March 2017. Patients were identified through review of electronic medical records and aPTT-based CWA parameters were retrieved from our laboratory data storage system retrospectively between two to five weeks after their initial presentation.

### Subjects

A total of 45 patients with acute VTE were included in this study. The inclusion criteria were: 1) objectively confirmed VTE based either on ultrasound or computed tomography (CT) scan findings with clinical features consistent with acute VTE; 2) a baseline aPTT performed at diagnosis and prior to the initiation of anticoagulation treatment. For subgroup analysis, acute VTE cases were further subclassified into provoked (N = 36) and unprovoked VTE (N = 9) cases. The provoked cases were subdivided accordingly into cancer associated thrombosis (N = 19) and non-cancer associated thrombosis (N = 17). The control group included patients with a baseline pre-operative aPTT performed as this was used for the purpose of this study. A total of 111 controls were included in this study. Study subjects and controls were excluded if they had a VTE occurring within the previous three months, were receiving any anticoagulant and were having an active infection. In addition, control subjects must not have had active cancer or ongoing treatment with chemotherapy or hormonal therapy for cancer.

#### Blood sampling

Blood samples were collected into 2.7 mL vacuum tubes containing 3.2% sodium citrate (Becton-Dickinson Company, New Jersey, USA) for aPTT analysis. The samples were centrifuged (Andreas Hettich GmbH & Co. KG, Tuttlingen, Germany) at 1500xg for 15 minutes. Platelet-poor plasma (PPP) was obtained with a verified platelet count of less than 10 x10^9^/L. Dade Actin FSL reagent (Siemens Healthcare, Marburg, Germany) containing purified soy phosphatides and rabbit brain phosphatides in 1.0 x 10^-4^M ellagic acid was used for the determination of aPTT within one hour of collection. Activated partial thromboplastin time was analysed using the Sysmex CS2100i automated coagulation analyser (Sysmex Corporation, Kobe, Japan). Samples with haematocrit exceeding 55% and haemolysed samples were not included in the analysis. The assay variation in our laboratory is maintained below 5%.

#### Collection of CWA data and outcome measures

**Figure 1:**
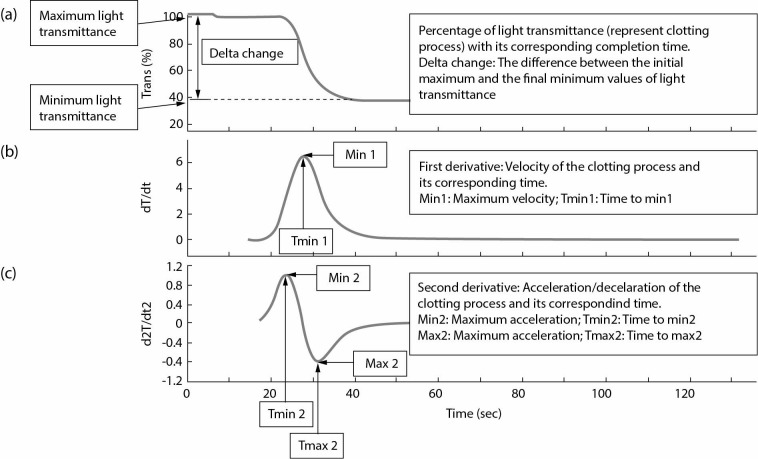
A representative clot waveform curve, its analysis and parameters. (a) The original transmittance curve obtained from the routine aPTT test. (b) The first derivative curve derived from the original transmittance curve. (c) The second derivative curve derived from the first derivative curve.

From the original transmittance curve, the first derivative curve, which describes the velocity of the clotting process, is obtained with the maximum velocity termed as min1 (Figure 1). The second derivative curve is in turn derived from the first derivative curve and describes the acceleration and deceleration of clot formation. Maximum acceleration is named min2 while maximum deceleration is called max2. Delta change is derived from the difference between the starting and final light transmittance levels. As the end point of the test is fibrin formation, delta change correlates to fibrin concentration and represents to some extent fibrin thickness as well as clot density (*18*). The first and second derivatives of the aPTT clot waveform have been postulated to represent thrombin and prothrombinase activities respectively (*16*). These aPTT-based CWA parameters were obtained from a built-in algorithm in the analyser which generated the clotting curve at 660 nm. Delta change however had to be calculated manually (Figure 1).

Defined outcome measures were the subjects’ aPTT, aPTT ratio, min1, min2, max2 and delta change values. The aPTT ratio was established using the subjects’ aPTT over the mean aPTT of healthy volunteers and an arbitrary ratio of less than or equal to 0.9 was determined to be shortened based on published data (*4*–*7*). The odds ratio for VTE was based on comparison with our laboratory’s reference intervals for CWA parameters.

#### Reference intervals

The reference intervals for aPTT and CWA parameters were established by our laboratory based on samples from 191 healthy volunteers in accordance with the Clinical and Laboratory Standards Institute (CLSI) Guidelines EP28-A3C. The mean and reference intervals were as follows: aPTT – 29 s (26 – 32), min1 – 4.77%/s (3.12 – 6.87), min2 – 0.75%/s^2^ (0.51 – 1.05), max2 – 0.62%/s^2^ (0.40 – 0.91) and delta change – 46.55% (29.11 – 71.63).

### Statistical Analysis

As there are no prior studies that compared CWA parameters between patients with VTE and controls, we used the Cohen’s standardized effect size to estimate the sample size required (*19*). A ratio of 1 VTE to 2 controls was used due to the small number of acute VTE patients with valid CWA parameters. In order to detect a moderate effect size (*i.e.* Cohen’s d = 0.5) with 80% power, 45 VTE patients and 90 controls were required. Eventually 45 VTE patients and 111 control patients were included in the study.

Normality testing was performed for all continuous variables using the Shapiro-Wilk test and presented as mean ± 2 SD or median (range) for normally distributed and skewed data respectively. Categorical variables were reported as N (proportion). The characteristics of patients and controls were compared using two-sample t-test or Mann-Whitney U test, and chi-square or Fisher’s exact test for categorical variables. The CWA parameters were compared between groups using two-sample t-test or Mann-Whitney U test, followed by multiple linear regression to adjust for differences in age, gender and ethnicity. Binary logistic regression models were used to analyze the associations of aPTT ratio and CWA parameters with VTE. In addition, the performance of each CWA parameter and combination of all four parameters were evaluated for prediction of VTE using receiver operating characteristic (ROC) analysis. The CWA parameters were first dichotomized into less than and equals to or more than the upper limit of their respective reference intervals. The binary logistic regression models were used to generate predicted probabilities of each CWA parameter being equals to or more than the upper normal limits. The predicted probabilities were then used in ROC analysis and the area under the curve (AUC) were reported. The sensitivity, specificity, positive and negative predictive values of the best performing CWA parameter from the ROC analysis were also reported. All analyses were performed using IBM SPSS Statistics for Windows, version 24 (IBM Corp., Armonk, USA) and P values < 0.05 were considered statistically significant.

## Results

The baseline characteristics of patients with acute VTE and control patients are shown in Table 1. Age, gender and racial distributions were not significantly different between the VTE and control groups.

**Table 1:** Baseline demographic characteristics of study subjects

**Demographics**	**VTE** **(N = 45)**	**Control** **(N = 111)**	**P**
Age, years	65 (22 – 89)	67 (25 – 84)	0.811
Gender, N (proportion)			0.384
Male	20 (0.44)	41 (0.37)	
Female	25 (0.56)	70 (0.63)	
Ethnicity, N (proportion)			
Chinese	34 (0.76)	97 (0.87)	0.075
Malay	8 (0.18)	6 (0.5)	
Indian	3 (0.7)	6 (0.5)	
Others	0 (0)	2 (0.2)	
VTE types, N (proportion)			
DVT only	20 (0.44)	/	/
DVT and PE	5 (0.11)	/	
PE only	13 (0.29)	/	
*Others	7 (0.13)	/	
Age is presented as median (range). VTE – venous thromboembolism. DVT – deep vein thrombosis. PE – pulmonary embolism. *Others included thrombosis of splenic, internal jugular, brachial, renal and portal veins. P < 0.05 was considered statistically significant.			

While the aPTT was not significantly different between VTE patients and controls, CWA parameters were significantly increased among patients with VTE (Table 2). Consequently, there were significantly more cases in the VTE group exhibiting CWA values above the upper limit of the respective reference interval for all four CWA parameters (min1, min2, max2 and delta change) compared to controls (Table 2). Having a CWA value above the upper limit of the respective reference interval carried the odds ratios for VTE of 8.0, 5.2, 4.8 and 18.6 for min1, min2, max2 and delta change, respectively (Table 3). In contrast, having an aPTT ratio of 0.9 or less carried only an odds ratio of 1.8 for VTE.

**Table 2:** Activated partial thromboplastin time, aPTT ratio and clot waveform analysis parameters

**Results**	**VTE** **(N = 45)**	**Control** **(N = 111)**	**P**
aPTT, s	28 ± 7	28 ± 3	0.830
aPTT ratio	1.0 ± 0.2	1.0 ± 0.1	0.830
aPTT ratio ≤ 0.9, N (proportion)	11 (0.24)	17 (0.15)	0.178
CWA parameters			
Min1, %/s	6.95 ± 3.02	5.54 ± 2.32	< 0.001
Min2, %/s^2^	1.09 ± 0.50	0.89 ± 0.38	0.001*
Max2, %/s^2^	0.89 ± 0.42	0.74 ± 0.32	0.002*
Delta change, %	68.44 ± 32.86	51.42 ± 23.40	< 0.001
CWA > ULRR, N (proportion)			
Min1 > 6.87%/s	25 (0.56)	15 (0.14)	< 0.001
Min2 > 1.05%/s^2^	26 (0.58)	23 (0.21)	< 0.001
Max2 > 0.91%/s^2^	20 (0.44)	16 (0.14)	< 0.001
Delta change > 71.63%	21 (0.47)	5 (0.5)	< 0.001
aPTT - activated partial thromboplastin time. aPTT and CWA parameters results are given as mean ± 2 standard deviations. VTE – venous thromboembolism. CWA – clot waveform analysis. ULRR - upper limit of reference interval. Proportions of cases within the venous thromboembolism (VTE) and control groups expressing aPTT ratio ≤ 0.9 and CWA parameters above the upper limit of the respective reference intervals are shown. aPTT ratio was established using the subjects’ aPTT over the mean aPTT of healthy volunteers. Reference intervals were established locally based on 191 healthy controls in accordance with the Clinical and Laboratory Standards Institute guidelines. *P-values < 0.001 after adjustment for age, gender and ethnicity. P<0.05 was considered statistically significant.			

**Table 3:** The association between aPTT and clot waveform analysis parameters and acute venous thromboembolism

	**Unadjusted**			Adjusted*		
**Parameters**	**OR**	**95% CI**	**P**	**OR**	**95% CI**	**P**
**aPTT ratio ≤ 0.9**	1.79	0.76 – 4.20	0.182	2.03	0.83 – 4.95	0.119
**Min1 > 6.87%/s**	8.00	3.59 – 17.83	< 0.001	8.37	3.63 – 19.26	< 0.001
**Min2 > 1.05%/s^2^**	5.24	2.48 – 11.07	< 0.001	5.93	2.68 – 13.11	< 0.001
**Max2 > 0.91%/s^2^**	4.75	2.15 – 10.48	< 0.001	4.99	2.20 – 11.34	< 0.001
**Delta change > 71.63%**	18.55	6.36 – 54.15	< 0.001	19.09	6.31 – 57.69	< 0.001
OR – odds ratio. CI – confidence interval. *Adjusted for age, gender and ethnicity. P < 0.05 was considered statistically significant.						

**Figure 2:**
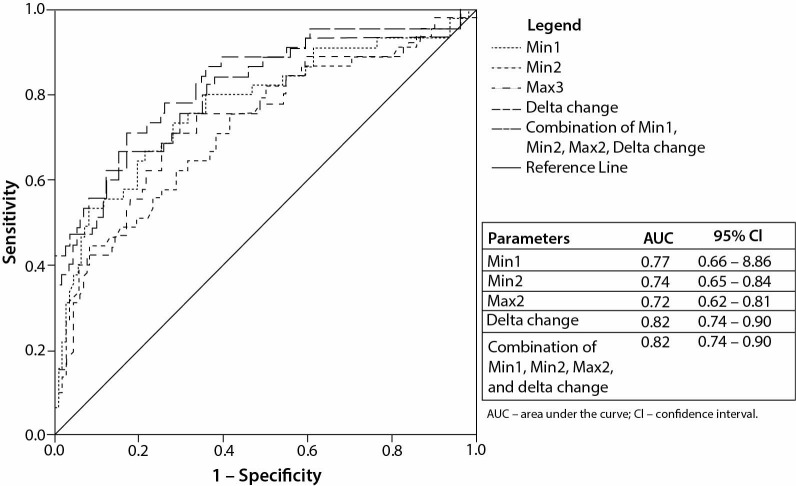
Receiver operating characteristics (ROC) curves and area under the curve (AUC) of the various CWA parameters (min1, min2, max2 and delta change individually as well as the composite of all these four markers) derived from logistic regression models using the upper limit of the reference interval of each CWA parameter. CWA - clot waveform analysis.

Using ROC analysis, all our measured CWA parameters displayed AUCs of greater than 0.70 (Figure 2). A delta change of 71.63% corresponding to the upper limit of our reference interval has the best AUC of 0.82. Using this delta change cut-off conferred a sensitivity of 47% and specificity of 93%. The positive and negative predictive values were 72% and 81%, respectively.

In subgroup analysis of provoked VTE cases, although the aPTT and CWA parameters did not differ, after adjustment for age, gender and ethnicity, CWA parameters (min1, min2 and max2) showed significant difference between cancer-associated and non-cancer-associated thrombosis cases (Table 4).

**Table 4:** Clot waveform analysis parameters of subset of patients with provoked acute venous thromboembolism

**Parameters**	**VTE with cancer** **(N=19)**	**VTE without cancer** **(N=17)**	**P**	Adjusted P*
**aPTT, s**	28 ± 9	28 ± 4	0.833	0.318
**Min1, %/s**	6.70 ± 3.10	7.62 ± 2.66	0.066	0.030
**Min2, %/s^2^**	1.06 ± 0.52	1.20 ± 0.46	0.081	0.033
**Max2, %/s^2^**	0.86 ± 0.42	0.98 ± 0.42	0.080	0.034
**Delta change, %**	67.94 ± 35.94	73.11 ± 26.60	0.338	0.163
Values are given as mean ± 2 standard deviations. VTE – venous thromboembolism. *P-values after adjustment for age, gender and ethnicity. P < 0.05 was considered statistically significant.				

## Discussion

This study provides new insights into the potential utility of CWA in detecting hypercoagulability in patients with acute VTE. Our findings showed that these patients have significantly higher CWA parameters than controls and values above the upper limit of their respective reference intervals were independently associated with acute VTE. Although it assesses the coagulation function of intrinsic and common pathways, the absolute aPTT value does not distinguish between patients with and without VTE. A shortened aPTT expressed as a ratio of the absolute clot time, however has previously been shown to be an independent risk factor for VTE with an odds ratio of approximately 2 (4-7). In our study, using a predetermined aPTT ratio of less than or equal to 0.9, we demonstrated the odds ratio for VTE was a more modest 1.79. In contrast, CWA parameters above the upper limit of their respective reference intervals yielded much higher odds ratios for acute VTE. This remained significantly associated with acute VTE even after accounting for differences in age, gender and ethnicity. CWA parameters may therefore offer a novel approach to assess patients’ risk of having acute VTE and hypercoagulability beyond what routine aPTT could offer. A recent report of a positive association between CWA and hypercoagulability using the Padua Prediction Score as a surrogate, among hospitalized medical patients further supports these findings (*17*).

Nonetheless, elevation of CWA parameters above the upper limit of their respective reference intervals only occurred in about 50% of patients with acute VTE. It was also not specific to patients with VTE as a minority of controls also had results exceeding the reference range. However, this is not unexpected since the hypercoagulable state as defined by CWA parameters is only one component of pathological thrombus formation and not a marker of formed thrombus.

Although CWA parameters have been shown to have statistically significant correlation with fibrinogen concentration, the strength of this relationship is at best moderate (*20*). This suggests that other aspects of the fibrin clot such as fibrin thickness and clot density may influence CWA. Furthermore, hyperfibrinogenemia has not been unequivocally demonstrated as a strong risk factor for VTE in contrast to alterations of fibrin clot properties that appear to have more important association with VTE and its recurrence (*21*, *22*). Although our present study does not shed new light on these observations, it further supports the contention that the hypercoagulable state is more than just the function of increased fibrinogen concentration alone.

In view of its strong positive association with acute VTE, we conducted further analysis to evaluate the potential of CWA as a diagnostic screening test for acute VTE. The AUC and the respective 95% confidence intervals showed that all the CWA parameters have similar discriminatory pattern and equally contribute to the better diagnostic performance. It is well-established that ELISA-based D-dimer assays have high sensitivity for VTE (> 95%) and hence high negative predictive value in excluding acute VTE especially in the presence of low pre-test clinical probability. However, the accompanying specificity is approximately 40% to 50%, rendering it ineffective as a “rule-in” test for VTE (*23*-*26*). In contrast, CWA parameters have demonstrated better specificity in our patients. This may be of value in our centre where approximately 0.5% of hospitalized patients have VTE (*27*). Using a single value of delta change greater than the upper limit of reference interval, this has a specificity in excess of 90% and a positive predictive value of more than 70%. This suggests that CWA could be a potentially useful marker in the diagnosis of acute VTE. As our study was not designed to address this, the definitive utility of CWA can only be confirmed in future larger prospective case control study. Our findings should provide the nidus for further investigations in this area.

While there is evidence of increased thrombin generation among patients with cancer associated thrombosis compared with healthy controls, comparison using global haemostatic assays between VTE patients with and without cancer was previously lacking. We have now provided some interesting data into this in our subgroup analysis. Expectedly, both groups exhibited higher CWA parameters than healthy controls. Although both groups did not demonstrate significant differences for their baseline aPTTs and CWA parameters, VTE patients with cancer had significantly lower CWA parameters than those without cancer following adjustment for age, gender and ethnicity. As aPTT-based CWA measures the global haemostatic function in plasma, our findings suggest that the hypercoagulability in patients with cancer associated thrombosis is contributed by other non-plasmatic factors. As such, patients with cancer might require lesser increment in plasmatic factors to form thrombus compared with patients without cancer. This is consistent with existing literature that suggest that various non-plasmatic factors are heightened in cancer patients contributing to the overall hypercoagulable state. These factors include platelets, microvesicles, leucocytes, activated endothelial cells and tumour cells (*28*). Nonetheless, due to the sample size limitation in this analysis, our findings will need to be validated in larger prospective studies.

The findings of this study should however be interpreted with consideration of the following limitations. As the haemolysed aPTT samples were excluded based only on visual examination, we might have inadvertently included some samples with minor level of haemolysis and this might confound some of our findings. As a marker for acute thrombosis, the specificity of CWA parameters has not been fully defined. Various innate and acquired conditions such as infections and medications can potentially influence CWA parameters and cannot be controlled because of limited available information. Our control population were patients admitted for elective surgery, which may not be representative of conditions that can confound the interpretation of our results. To serve as a reliable predictor of acute VTE, aPTT-based CWA parameters will need to be evaluated prospectively in patients at risk of VTE rather than the current retrospective analysis among patients with confirmed VTE. Comparison with other coagulation and haemostatic assays was also lacking in this present study. Lastly, the wide confidence intervals for the odds ratios obtained in this study reflect our relatively small sample and underpins the need for larger studies to verify our findings.

In conclusion, we have shown that elevated aPTT-based CWA parameters were associated with acute VTE and may have clinical utility in the diagnosis and prediction of acute VTE.
